# Potential Neuroprotective Role of Calretinin-N18 and Calbindin-D28k in the Retina of Adult Zebrafish Exposed to Different Wavelength Lights

**DOI:** 10.3390/ijms24021087

**Published:** 2023-01-06

**Authors:** Caterina Porcino, Marilena Briglia, Marialuisa Aragona, Kamel Mhalhel, Rosaria Laurà, Maria Levanti, Francesco Abbate, Giuseppe Montalbano, Germana Germanà, Eugenia Rita Lauriano, Alessandro Meduri, Josè Antonio Vega, Antonino Germanà, Maria Cristina Guerrera

**Affiliations:** 1 Zebrafish Neuromorphology Lab, Department of Veterinary Sciences, University of Messina, 98168 Messina, Italy; 2 Department of Chemical, Biological, Pharmaceutical and Environmental Sciences, University of Messina, Viale F. Stagno d’Alcontres 31, 98166 Messina, Italy; 3 Unit of Ophthalmology, Department of Biomedical and Dental Sciences, and of Morphological and Functional Images, University of Messina, 98124 Messina, Italy; 4 Departamento de Morfología y Biología Celular, Grupo SINPOS, Universidad de Oviedo, 33006 Oviedo, Spain; 5 Facultad de Ciencias de la Salud, Universidad Autónoma de Chile, Santiago 7500912, Chile

**Keywords:** CaBPs, retina, zebrafish

## Abstract

The incidence rates of light-induced retinopathies have increased significantly in the last decades because of continuous exposure to light from different electronic devices. Recent studies showed that exposure to blue light had been related to the pathogenesis of light-induced retinopathies. However, the pathophysiological mechanisms underlying changes induced by light exposure are not fully known yet. In the present study, the effects of exposure to light at different wavelengths with emission peaks in the blue light range (400–500 nm) on the localization of Calretinin-N18 (CaR-N18) and Calbindin-D28K (CaB-D28K) in adult zebrafish retina are studied using double immunofluorescence with confocal laser microscopy. CaB-D28K and CaR-N18 are two homologous cytosolic calcium-binding proteins (CaBPs) implicated in essential process regulation in central and peripheral nervous systems. CaB-D28K and CaR-N18 distributions are investigated to elucidate their potential role in maintaining retinal homeostasis under distinct light conditions and darkness. The results showed that light influences CaB-D28K and CaR-N18 distribution in the retina of adult zebrafish, suggesting that these CaBPs could be involved in the pathophysiology of retinal damage induced by the short-wavelength visible light spectrum.

## 1. Introduction

The most recent WHO estimates suggest that, in the world, 285 million people suffer from visual disabilities and, of these, 39 million are blind. The prevalence of people affected by blindness and vision impairment is predicted to increase and reach a value of 115 million by 2050 [1,2]. Risk factors predispose to the onset of ocular pathogenesis, among them oxidative stress, vascular changes, and light radiation. This latter can damage the eye through photomechanical, photochemical, and photothermal mechanisms [3,4]. Photochemical retina injury induced by light exposure depends on light wavelength. Short wavelength radiations are the most harmful. In the last few years, human exposure to UV light has increased because of the constant exposure to several electronic devices that emit up to 40% light in the wavelength range between 400 to 490 nanometers, causing irreparable damage to the retina [5]. Cytotoxicity blue light-induced was demonstrated [6], in particular, photoreceptor injury was observed by Tosini et al. [7]. Light stimuli affect calcium cell concentration [8] and an altered balance of this ion contributes to the appearance of degenerative processes in the retina [9] since calcium is involved in cell development and maturation, synaptogenesis, and maintenance of neuronal circuits in this organ [10,11]. Calcium homeostasis is regulated by calcium-binding proteins (CaBPs), including the EF–hand proteins Calretinin-N18 (CaR-N18) and Calbindin (CaB-D28K). These proteins are widely distributed in central and peripheral nervous systems, including the retina [12,13,14,15] where they are employed as neuronal markers [16,17,18,19,20,21,22]. CaR-N18 and CaB-D28K are involved in calcium signaling regulating message targeting, intracellular calcium buffering, and neuronal excitability modulation [23,24,25,26,27,28]. Presumably, CaB-D28K can play a role in retinal disease. However, its precise roles in retinal disease are still unknown and remain the object of further studies. It was suggested that these calcium-binding proteins could also have a protective role in the retina [29]. CaB-D28K has been attributed to a protective role against the horizontal cells and ganglion cells of rats with retinal ischemia. Further, CaBPs can protect against excitotoxicity caused by an increased release of neurotransmitters by regulating the concentration of intracellular calcium [30,31,32,33,34,35,36,37,38,39]. Both mammals and fish, including zebrafish (*Danio rerio*), express CaR-N18 and CaB-D28K [40,41,42,43,44,45]. Zebrafish is commonly used in biomedical research to study human pathologies, including visual disorders [46,47,48,49] and photochemical stress, because it shows all the characteristics are considered suitable for scientific research [50,51]. In zebrafish and other vertebrates, the expression of the different CaBPs in the retinal neurons was studied, but their role is still unclear. Moreover, it was never investigated if light exposure could affect CaR-N18 and CaB-D28K distribution in the retina. Therefore, this study intended to compare the immunohistochemical localizations of CaR-N18 and CaB-D28K in the retina of adult zebrafish exposed to different wavelengths of light to understand if light could affect the pattern of distribution of these proteins. Finally, the present study could provide additional information on the potential involvement of CaR-N18 and CaB-D28K in retinal homeostasis maintenance.

## 2. Results

**Figure 1:**
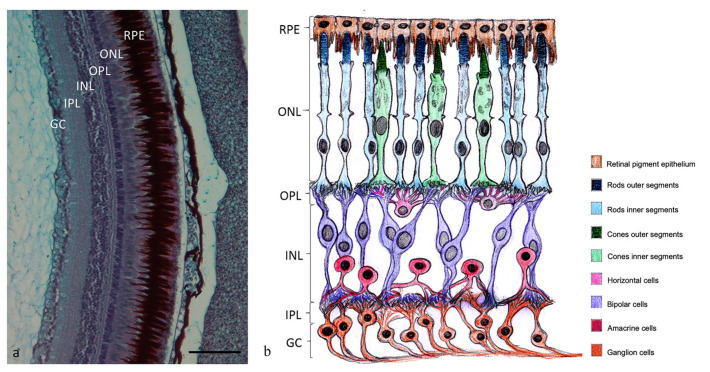
(**a**) Retina of adult zebrafish: RPE, retinal pigment epithelium; ONL, outer nuclear layer; OPL, outer plexiform layer; INL, inner nuclear layer; IPL, inner plexiform layer; GC, ganglion cell layer; Alcian Blue-PAS staining. Magnification 20×. (**b**) Graphical representation of the cell layers into the retina.

The zebrafish retina shows an overlapping stratigraphy to mammals, including humans (Figure 1).

**Figure 2:**
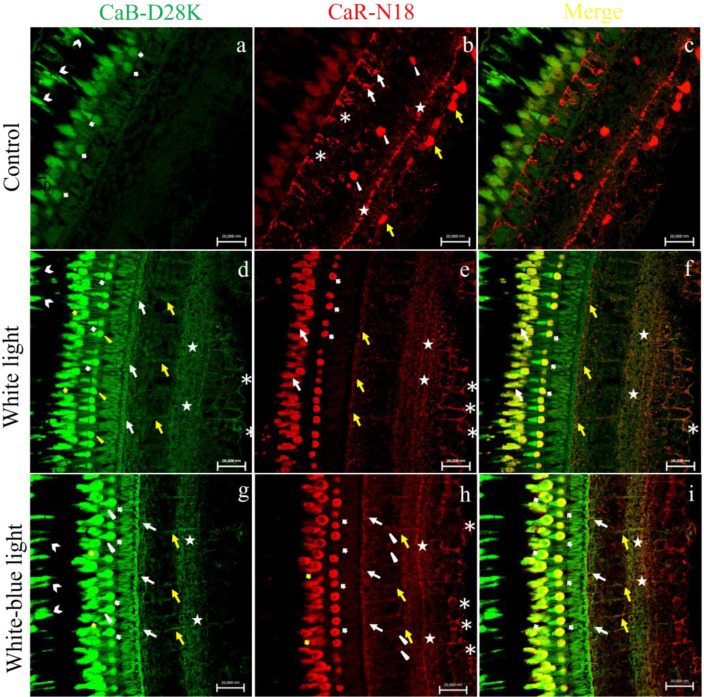

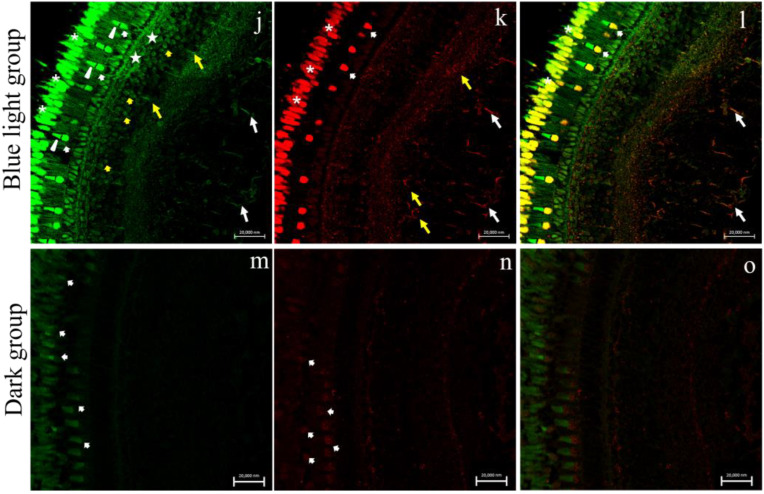
Calbindin-D28K/Calretinin-N18 immunostaining in zebrafish (*Danio rerio*) retina, approximately 4-months-old. (**a**–**c**) Calbindin-D28K/Calretinin-N18 immunostaining in control group (natural photoperiod conditions) (**a**) CaB-D28K-immunoreactivity in the cytoplasmic prolongations of the cells of the retinal pigment epithelium (RPE) (chevron arrows), in the cones and rods of the photoreceptors layer (bold arrows). (**b**) CaR-N18 immunoreactivity in the outer plexiform layer (OPL) (asterisks), in the soma of a subpopulation of amacrine cells (arrowheads) and bipolar cells (arrows), in the inner plexiform layer (IPL) (stars), and in the soma of ganglion cells (GCs) (yellow arrows). (**c**) Merge: absence of Calbindin-D28K/Calretinin-N18 colocalization. (**d**–**f**) Calbindin-D28K/Calretinin-N18 immunostaining in white light group (emission 34.8% of 400–500 nm). (**d**) CaB-D28K-immunoreactivity in the cytoplasmic prolongations of the cells of the retinal pigment epithelium (RPE) (chevron arrows), in the outer segments of cones (bold arrows), and inner segment of cones (yellow arrowheads) and in the rods (yellow bold arrows), in the outer plexiform layer (OPL) (arrows), in the bipolar cells (yellow arrows), in the inner plexiform layer (IPL) (stars), and in the ganglion cells and their axons (GC) (asterisks). (**e**) Calretinin-N18 immunoreactivity in the inner segments of cones (bold arrows), in the rods (arrows), in the innermost layer of the OPL (yellow arrows), in the innermost layer of the IPL (stars) and in the GCs and their axons (asterisks). (**f**) Merge: Calbindin-D28K/Calretinin-N18 colocalization in inner segments of cones (bold arrows), in the rods (arrows), in the innermost layer of IPL (stars) and OPL (yellow arrows) and in the GCs and their axons (asterisks). (**g**–**i**) Calbindin-D28K/Calretinin-N18 immunostaining in white–blue light group (emission 54.6% of 400–500 nm). (**g**) Calbindin-D28K immunoreactivity in retinal pigment epithelium (RPE) into the cytoplasmic prolongations of its cells (chevron arrow), in the inner (bold arrows) and outer segment of cones (arrows heads), in the rods (yellow bold arrows), in the innermost layer of OPL (arrows), in the bipolar cells (yellow arrows) and in the IPL (stars). (**h**) Calretinin-N18 immunoreactivity in the inner segments of cones (bold arrows), in the rods (yellow bold arrows), in the innermost layer of OPL (arrows), in the BCs (yellow arrows), in a subpopulation of amacrine cells (arrowheads), in the innermost layer of OPL (stars), and in the GCs (asterisk). (**i**) Merge: Calbindin-D28K/Calretinin-N18 colocalization in the photoreceptor layer (bold arrows), in the innermost layer of OPL (arrows), in the BCs (yellow arrows) and in the innermost layer of IPL (stars). (**j**–**l**) Calbindin-D28K/Calretinin-N18 immunostaining in blue light group (emission 84.3% of 400–500 nm). (**m**) Calbindin-D28K immunoreactivity in the outer (arrowheads) and inner (bold arrows) segment of cones and in the rods (asterisks), in the OPL (stars), in some amacrine cells (yellow bold arrows), in the BCs (yellow arrows), and GCs (arrows). (**n**) Calretinin-N18 immunoreactivity in the inner segments of cones (bold arrows) and rods (asterisks), in a subpopulation of amacrine cells (yellow arrows), GCs, and their axons (arrows). (**o**) Merge: Calbindin-D28K/Calretinin-N18 colocalization in the inner segments of cones (bold arrows) and in the rods (asterisks) and in some GCs and their axons (arrows). (**m**–**o**) Sample kept in dark conditions. (**m**) Low CaB-D28K immunoreactivity and (**n**) low CaR-N18 immunoreactivity in the photoreceptors layer. No immunostaining to Calbindin-D28K (**a**) and Calretinin-N18 (**b**) in the other retinal layers. (**o**) Merge: no immunostaining to Calbindin-D28K/Calretinin-N18. Magnification 40× (**a**–**o**).

The comparison of the immunohistochemical localization of Calbindin (CaB-D28K) and Calretinin (CaR-N18) revealed distinct distribution patterns in the retinal layers in the different experimental groups. In the control group samples (kept in natural photoperiod), retinal pigment epithelium (RPE) showed a CaB-D28K immunoreactivity in the cytoplasmic prolongations of its cells (microvilli). CaB-D28K-immunoreactivity was observed in the photoreceptors layer, both in cones and rods. CaR-N18 immunoreactivity was observed in the outer plexiform layer, in the soma of a subpopulation of amacrine cells and bipolar cells, in the inner plexiform layer (IPL), and in the soma of ganglion cells (GCs) (Figure 2a–c). White light-exposed samples showed a CaB-D28K-immunoreactivity in the RPE cytoplasmatic prolongations and both in the outer and inner segments of cones and rods. A CaB-D28K-immunoreactivity was found in the outer plexiform layer (OPL), in the bipolar cells (BC), in the inner plexiform layer (IPL), and the ganglion cells and their axons (GC). CaR-N18-immunoreactivity was found in the inner segment of cones, in the rods, in the innermost layer of the OPL, in the innermost zone of the IPL, and the ganglion cells and their axons (GC). A CaB-D28K/CaR-N18 colocalization was observed in the inner segments of cones and outer segments of rods, in the IPL, and the GCs and their axons (Figure 2d–f). Sample white–blue light-exposed showed a CaB-D28K-immunoreactivity in the cytoplasmic prolongations of the RPE, in the outer segment of cones, in the rods, in the OPL, in BCs, and in IPL. A CaR-N18 immunoreactivity was found in the inner segment of cones, in the rods, in the innermost of OPL, in the BCs. A low immunoreactivity to CaR-N18 was found in a subpopulation of amacrine cells. Finally, the innermost layer of IPL and GCs and their axons showed CaR-N18 immunoreactivity also. CaB-D28K/CaR-N18 colocalization was found in the inner segment of cones, in the rods, in the innermost layer of the OPL, in the BCs, and in the IPL (Figure 2g–i). A sample exposed to blue light showed CaB-D28K-immunoreactivity in the outer and inner segments of cones and in the rods. A CaB-D28-immunoreactivity was found in the OPL, bipolar cells, some amacrine cells, and ganglion cells and their axons. The inner segments of cones and rods, a subpopulation of amacrine cells, ganglion cells, and their axons were immunoreactive to CaR-N18. CaB-D28K and CaR-N18 were colocalized in the inner segments of cones and in the rods, and in some ganglion cells and their axons (Figure 2j–l). Samples held in darkness showed low CaB-D28K and CaR-N18 immunoreactivity in the photoreceptors layer, but there was no immunoreactivity in the other retinal layers (Figure 2m–o).

## 3. Discussion

Despite its peripheral location, the retina or neural portion of the eye is part of the central nervous system, and it is continuously exposed to light radiation. As mentioned above, it is widely recognized that long-term exposure to high-intensity light often causes retinal lesions. It has been demonstrated that short-wavelength visual blue light negatively affects mitochondrial function, causing oxidative stress and decreased cell survival [52,53]. Moreover, Sanchez et al. [54] demonstrated that light regulates the expression of the BDNF and its receptor TrkB in the retina of adult zebrafish under a short-wavelength visible light spectrum. The present study is conducted on adult zebrafish because its retina shows interesting morpho-functional similarities with the human retina. For instance, they share the density of cones and the presents of rods, and light-perception mechanism. Moreover, humans and zebrafish show an overlapping stratigraphy of the retina, consisting of three cell layers interspersed with two synaptic layers [55,56,57,58]. For all these reasons, zebrafish turned into a valuable model for studying many vision disorders compared to the more traditional mouse model [59,60,61,62]. Calbindin (CaB-D28K) and Calretinin (CaR-N18) are calcium-related proteins in the retina and are mainly distributed in the neuroepithelial layer. Their decrease leads to disorders of calcium-dependent activities. At the same time, the decreased expression of CaB-D28K and CaR-N18 leads to the dysfunction of horizontal, amacrine, and bipolar cells in the retina, reducing visual function [41,63]. With the present investigation, we detect the presence of CaB-D28K in the cytoplasmatic prolongation of the RPE. This is intriguing because this epithelium plays a vital role in keeping rods and cones healthy and well-functioning. The RPE is involved in the absorption of light passing through the neural retina, and it participates in the restoration of photosensitivity of dissociated visual pigments in response to light [64]. In control samples, CaB-D28K and CaR-N18 distribution is similar to their distribution in the human retina. For instance, CaB-D28K is expressed in the cones of both zebrafish and humans [65,66]. On the other hand, CaB-D28K is not expressed in the cones of the retina of the rat and mouse [67]. This could be a piece of evidence confirming the zebrafish suitability as a model organism in retinal disorders studies in translational medicine. Zebrafish, humans, mice, and rats showed CaR-N18-immunoreactivity in a subpopulation of amacrine cells [25,68]. In the zebrafish retina, bipolar cells are CaR-N18-immunoreactive, while in bipolar cells of humans, CaR-N18 is colocalized with CaB-D28K [69,70]. On the contrary, no immunoreactivity to CaR-N18 was found in bipolar cells of mice and rats [25,68]. In the same way as rats and humans, zebrafish show immunoreactivity to CaR-N18 in its ganglion cells [43,68,69,71]. On the contrary, the GCs of mice show no immunoreactivity to CaR-N18 [71]. For a comparison of different species CaB-D28K/CaR-N18 localization in the retina layers, see Table 1. Although the expression of CaB-D28K and CaR-N18 in developing and regenerating zebrafish visual system was previously studied [9], their neuronal distribution after light exposure at different wavelengths has not yet been investigated. Some studies have considered the effects of red light on the localization of these CaBPs in the ischemic retina [72]. Hence, the present study demonstrates, for the first time, that the distribution of these two calcium-binding proteins (CaBPs) changes under distinct wavelength light stimuli and dark conditions.

### 3.1. White Light Experimental Group versus Control

**Figure 3:**
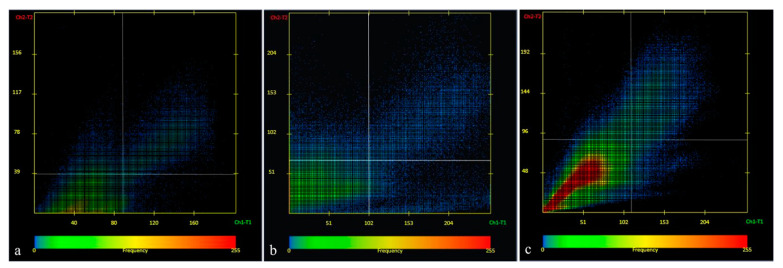
Scatter plot of CaB-D28K/CaR-N18 colocalization in different experimental groups: (**a**) white-light experimental group; (**b**) white/blue-light experimental group; (**c**) blue-light experimental group. Zen 2011 (LSM 700 Zeiss software, Germany).

Compared to the control, the white-light experimental group showed CaR-N18-immunoreactivity in the inner segment of cones and in the rods in colocalization with CaB-D28K (Figure 3a) that is also localized in the RPE, in the outer segments of cones, and in the outer plexiform layer. Moreover, in contrast to the control group, CaB-D28K immunopositivity was observed in the IPL, bipolar cells, and ganglion cells. CaR-N18-immunoreactivity was not found in the amacrine cells of the samples exposed to white light, contrary to what was detected in the control group samples. CaB-D28K-immunoreactivity was found in the outer plexiform layer, in the inner plexiform layer, contrary to what was detected in the control group samples, and in the ganglion cells in colocalization with CaR-N18.

### 3.2. White–Blue Light Experimental Group versus Control

In the same way as the control group, CaB-D28K is localized in the RPE of the white–blue light exposed sample. In this experimental group, a CaB-D28K and CaR-N18 colocalization was observed in the inner segment of cones (Figure 3b) and in the rods, whereas, in the control group, CaB-D28K was expressed alone in the inner segments of the rods and the inner and outer segments of the cones. In contrast to the control group, CaB-D28K was found in OPL, bipolar cells, and the IPL. Moreover, CaB-D28K-immunoreactivity appeared in colocalization to CaR-N18 in the OPL, bipolar cell, and IPL of the sample of this experimental group. On the contrary, bipolar cells were immunoreactive only to CaR-N18 in the control group. The CaR-N18-immunoreactivity of the OPL and IPL of the control group samples was also found in the OPL and IPL of the white–blue light-exposed samples. In comparison to the control, CaR-N18-immunoreactivity remained unchanged in ganglion cells. Amacrine cells of white–blue light exposed samples showed an immunoreactive to CaR-N18 but were lower than the control group.

### 3.3. Blue Light Experimental Group versus Control

In blue light-exposed samples, the CaB-D28K immunoreactivity is absent in the RPE, unlike the control samples. In this experimental group, CaR-N18 immunoreactivity appeared in colocalization with CaB-D28K in the inner segment of cones and in the rods (Figure 3c). Contrary to the control, CaB-D28K immunoreactivity was observed in OPL, bipolar cells, amacrine cells, and ganglion cells, and no CaR-N18-immunoreactivity was observed in the OPL of the sample of the experimental group. If on one hand CaR-N18 immunoreactivity remained unchanged with respect to control in amacrine and GCs of this experimental group, on the other, it disappeared in bipolar cells and IPL. This experimental group differs from the control one for the CaB-D28K/CaR-N18 colocalization in the GCs. Some authors assume that CaR-N18 has neuronal protection function because it is not expressed when visual system damage occurs [9]. This evidence could be related to the shutdown of the CaR-N18 fluorescent signal in the OPL and IPL of our blue-light experimental group.

### 3.4. Darkness Experimental Group versus Control

It is now well-established that light stimulation is essential for vertebrate retina maintenance during adulthood [89,90,91]. Hence, it could be speculated that long-term exposure to darkness made the retina of the samples of this experimental group less responsive to light, especially after a prolonged period (10 days). From a biochemical point of view, a possible explanation for the weakening of the fluorescent signal in photoreceptors and the lack of expression on the other retinal layer could be that darkness acts by inducing a release of inhibitory neurotransmitter and a block of Ca^2+^ in the storage site, so CaBPs presence is unnecessary. On the other hand, the localization of CaB-D28K in the cones of the samples held in the dark could be related to the nature of these photoreceptors involved in nocturnal vision mechanisms.

### 3.5. Comparison between the Distinct Experimental Groups

**Figure 4:**
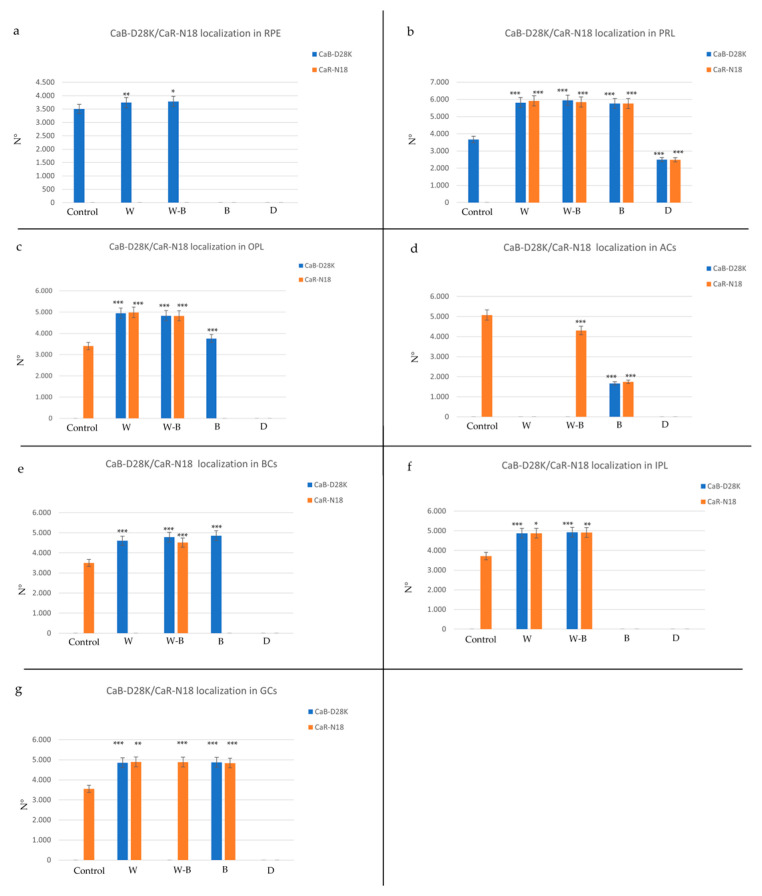
Graphical representation of immunopositivity of: (**a**) retinal pigment epithelium (RPE), (**b**) photoreceptor layer (PRL), (**c**) outer plexiform layer (OPL), (**d**) amacrine cells (ACs), (**e**) inner plexiform layer (IPL), (**f**) bipolar cells (BCs), (**g**) ganglion cells (GCs) detected by Calbindin (CaB-D28K) and Calretinin (CaR-18N) in different experimental groups. Experimental conditions: control (natural photoperiod), white light with a 34.8% of blue light emission (W), white–blue light with a 54.6% of blue light emission (WB), blue light with an 84.3% of blue light emission (B) and darkness (D). The statistical analysis shows a different distribution pattern of the two proteins between the experimental groups compared to the control. Data represent the average of measurements from the ten sections from each treatment. Statistical significance: *** *p* < 0.001, ** *p* < 0.01, * *p* < 0.05.

What emerges from our observations is that artificial light affects CaB-D28K and CaR-N18 distribution in the different retinal layers, regardless of the light wavelengths. As a matter of fact, the statistical analysis shows a different distribution pattern of the two proteins between the experimental groups compared to the control (natural photoperiod) (Figure 4 and Table 2). It is recognized that EF–hand CaBPs are involved in homeostasis and neuroprotection thanks to their Ca^2+^ buffering properties [8]. Therefore, the different distribution patterns of CaB-D28K and CaR-N18 could be related to an increased Ca^2+^ concentration in retinal cells due to the light stimuli. What is reported in the literature is that Calretinin-immunoreactivity changes in some pathological conditions of the retina, for instance, in amacrine cells [92]. This pattern is shared with the sample kept in white, white–blue, and dark conditions during our experimentation. A recurring trend regards the localization of Calbindin in the OPL of the retina of all artificial light-exposed animals. Considering that the neuroprotective role of Calbindin is recognized by some members of the scientific community [8,93], this trend could be interpreted as a response of some cells to protect themselves from excessive excitability induced by chronic exposure to different artificial lights. In the same way, Calbindin appears in all the bipolar cells of fish exposed to artificial light. Another recurring trend is the appearance of Calretinin in the cones and rods of the samples exposed to all the lights.

## 4. Materials and Methods

### 4.1. Experimental Protocol

**Figure 5:**
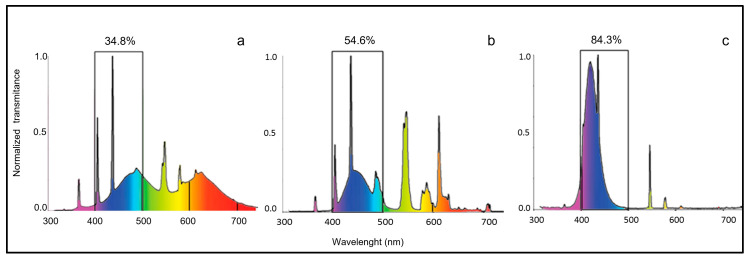
Spectral distribution of the illumination sources. (**a**) white light, (**b**) white–blue light, (**c**) blue light. The percentage of blue light emission for each experiment is indicated.

For this study, twenty adult zebrafish paraffin, embedded for previous experimentation, were used (see Sánchez Ramos et al. [54]). In that experiment, the groups were exposed to white light, white–blue light, blue light, and darkness, respectively, while the fishes of the control group were kept in natural photoperiod conditions. Experimental conditions had been maintained continuously for 10 days for all groups [54]. Fish have been maintained at 28 °C using standard conditions in different transparent plastic tanks. Light sources were placed 3 cm above the tank (for illumination source details, see Table 3 and Figure 5). Furthermore, a group of fish was exposed to darkness in a special black tank. The characteristics of each type of light, to which different experimental groups were exposed, were determined by the Department of Optics II, Universidad Complutense, Madrid, Spain. They were chosen to investigate the effects of the different percentages of blue light content. Four specimens for each of the five experimental groups were employed. The included tissue samples were then cut into 7 µm thick serial sections and collected on gelatin-coated microscope slides [94,95,96,97]. Some sections were deparaffinized and rehydrated, washed in distilled water, and stained with Alcian Blue pH 2.5 Periodic Acid Schiff (AB/PAS) (04-163802, Bio-Optica Milano S.p.a., Milan, Italy). Sections were examined under an OLYMPUS BX51 system Microscope (Olympus Optical Co., Ltd., Nagano, Japan), and micrographs were taken using a digital camera OLYMPUS DP12 (Olympus Optical Co., Ltd., Nagano, Japan).

### 4.2. Confocal Immunofluorescence

To analyze the expression of different proteins in zebrafish retina tissue, sections were deparaffinized and rehydrated, washed in Phosphate-Buffered Saline (PBS) 0.1 M pH = 7.4, and incubated in 0.3% H_2_O_2_ (PBS) solution for 3 min to prevent the activity of endogenous peroxidase; then, to rinsed sections, fetal bovine serum was added (F7524 Sigma-Aldrich, St. Louis, MO, USA). Calretinin goat antibody (N18-sc-11644 Santa Cruz Biotechnology, Santa Cruz, CA, USA) was used in double-label experiments with a monoclonal antibody to Anti-Calbindin-D28K (C9848 Sigma-Aldrich). Both the employed antibodies were diluted 1:100. Sections were incubated overnight at 4 °C in a humid chamber with antibodies. The specificity of the antibodies has been previously tested in zebrafish [13,98,99,100]. After rinsing in PBS, the sections were incubated for 1 h at 4 °C with Donkey anti-Goat IgG (H+L) Alexa Fluor 594 (Invitrogen A-11058, Waltham, MA, USA, 1:100); Donkey anti-Mouse IgG (H+L) Alexa Fluor 488 (Invitrogen A-21202 1:100). Both steps were performed at room temperature in a dark humid chamber. Finally, the section was washed and mounted with Fluoromount Aqueous Mounting Medium (Sigma Aldrich, USA) to prevent photobleaching, and then they were cover-slipped. To provide negative controls, representative sections were incubated with specifically preabsorbed antisera as described above. Under these conditions, no positive immunostaining was observed.

The immunofluorescence was detected using a Zeiss LSMDUO confocal laser scanning microscope with META module (Carl Zeiss Micro Imaging GmbH, Göttingen, Germany) microscope LSM700 AxioObserver. Zen 2011 (LSM 700 Zeiss software) built-in “colocalization view” was used to highlight the expression of both antibodies signals in order to produce a “colocalization” signal, the scatter plot, and fluorescent signal measurements. Each image was rapidly acquired to minimize photodegradation.

### 4.3. Statistical Analysis

ImageJ software was used to evaluate microscope fields collected randomly. One-way ANOVA was used to examine the statistical significance of the quantity of retinal pigment epithelium (RPE), photoreceptor layer (PRL), outer plexiform layer (OPL), amacrine cells (ACs), inner plexiform layer (IPL), bipolar cells (BCs), ganglion cells (GCs) detected by CaB-D28K, and CaR-18N in different experimental conditions. SigmaPlot version 14.0 (Systat Software, San Jose, CA, USA) was used to conduct the statistical analysis. An unpaired Z test was also performed. The information was given as median values with standard deviations (Δσ). Values of *p* below 0.05 were considered statistically significant in the following order *** *p* < 0.001, ** *p* < 0.01, * *p* < 0.05.

## 5. Conclusions

The present study demonstrates that light regulates Calbindin (CaB-D28K) and Calretinin (CaR-N18) distribution in the retina of adult zebrafish. It suggests that these calcium-binding proteins (CaBPs) could be involved in the pathophysiology of short-wavelength visible light spectrum retinal damage. However, it remains to be elucidated if the expression of Calbindin and Calretinin in cells where usually they are not expressed (and vice versa their silencing) could be intended as a protective response to light or if light-induced damage occurs through an altered localization of these two calcium-binding proteins. This kind of understanding could represent a starting point for future studies on phototoxicity, and retinal degeneration. To investigate the potential therapeutic role of CaBPs in the treatment of light-induced retinal damage, further studies are needed to elucidate the function of CaR-N18 and CaB-D28K in the recovery of normal conditions after retinal injury due to exposure to UV light and their role in ocular pathogenesis. In our laboratory, ultrastructural analysis of light-induced injury is underway in the adult zebrafish retina as a model for human ocular disorders. Finally, it is necessary to bring new knowledge to CaB-D28K and CaR-N18 role in retinal homeostasis to elucidate their potential role as markers of retinal damage for transversal studies. The findings of the present study demonstrate that CaBPs could be involved in the protection of neurons from light-induced neurodegeneration.

## Figures and Tables

**Table 1:** Comparison of different species CaB-D28K/CaR-N18 localization in the retina layers.

	Zebrafish *		Rat		Refs.	Mouse		Ref.	Human		Refs.
	CaB-D28K	CaR-N18	CaB-D28K	CaR-N18		CaB-D28K	CaR-N18		CaB-D28K	CaR-N18	
**RPE**	+	−		+	[68]				+		[69,70]
**PRL**	+	−	−	+	[68,73]	−		[74]	+	−	[25,65,67,73,75]
**OPL**	−	+	+	+	[73,76]	+		[77]	+	−	[73,78]
**INL**			+	+	[68,73,76,79,80]	+	+	[81,82]	+	+	[20,83]
**Bipolar cells**	−	+	+	−	[68,84]	−	−	[25]	+	+	[25,73,83]
**Amacrine cells**	−	+	+	+	[67,73,79,85,86]	+	+	[25,87]	+	+	[25,67,73,83]
**IPL**	−	+	+	+	[68,73,76,79,80]	−	+	[74,81,82,87]	+	+	[70,83,88]
**GCL**	−	+	+	+	[67,68,73,76,80,85,86]	+	+	[25,82,87]	+	+	[25,67,73,75,83]

Retinal pigment epithelium (RPE), photoreceptor layer (PRL), outer plexiform layer (OPL), inner plexiform layer (INL), inner plexiform layer (IPL), ganglion cell layer (GCL). * these data refer to the control group sample of the present study.

**Table 2:** Mean data ± standard deviation (∆σ) of immunopositivity of: GC (ganglion cell); IPL (inner plexiform layer); INL (inner nuclear layer); OPL (outer plexiform layer); ONL (outer nuclear layer); RPE (retinal pigment epithelium) detected by Calbindin (CaB-D28K) and Calretinin (CaR-18N). Experimental conditions: control (natural photoperiod), white light with a 34.8% of blue light emission (W), white–blue light with a 54.6% of blue light emission (WB), blue light with an 84.3% of blue light emission (B), and darkness (D). The statistical analysis shows a different distribution pattern of the two proteins between the experimental groups compared to the control. All features were evaluated per 174.286 ± 3.082 μm (mean). Statistical significance: *** *p* < 0.001, ** *p* < 0.01, * *p* < 0.05.

Treatment	Mean ± ∆σ in RPE		Mean ± ∆σ in PRL		Mean ± ∆σ in OPL		Mean ± ∆σ of Amacrine Cells		Mean ± ∆σ of Bipolar Cells		Mean ± ∆σ in IPL		Mean ± ∆σ of Ganglial Cells	
	CaB-D28K	CaR-N18	CaB-D28K	CaR-N18	CaB-D28K	CaR-N18	CaB-D28K	CaR-N18	CaB-D28K	CaR-N18	CaB-D28K	CaR-N18	CaB-D28K	CaR-N18
**Control**	3.503 ± 0.45	-	3.663 ± 0.73	-	-	3.403 ± 0.54	-	5.076 ± 0.66	-	3.503 ± 0.45	-	3.713 ± 0.58	-	3.553 ± 0.47
**White**	3.743 ± 0.30 **	-	5.810 ± 1.33 ***	5.910 ± 1.39 ***	4.946 ± 2.11 ***	4.986 ± 2.15 ***	-	-	4.602 ± 0.85 ***	-	4.876 ± 1.74 ***	4.876 ± 1.74 *	4.855 ± 1.73 ***	4.894 ± 1.52 **
**White–Blue**	3.786 ± 0.41 *	-	5.944 ± 1.42 ***	5.844 ± 1.34 ***	4.833 ± 1.37 ***	4.824 ± 1.36 ***	-	4.303 ± 0.66 ***	4.782 ± 0.62 ***	4.513 ± 0.58 ***	4.924 ± 1.51 ***	4.915 ± 1.26 **	-	4.888 ± 0.88 ***
**Blue**	-	-	5.755 ± 0.95 ***	5.515 ± 0.95 ***	3.758 ± 0.29 ***	-	1.668 ± 0.40 ***	1.746 ± 0.39 ***	4.855 ± 1.73 ***	-	-	-	4.874 ± 0.48 ***	4.838 ± 0.65 ***
**Dark**	-	-	2.500 ± 1.06 ***	2.485 ± 0.42 ***	-	-	-	-	-	-	-	-	-	-

**Table 3:** Illumination source details. Irradiancies were measured using a photoradiometric TektronixJ1800 (TekLumaColor, Wilsonville, OR, USA). The spectral composition of the light source was determined using the optic fiber spectrophotometer Spectrawiz EPP2000 (StellarNet, Keystone, FL, USA).

	White Light	White–Blue Light	Blue Light
**Source**	Philips MASTER TL-D Reflex 18W/840	RADIUM NL 18W/965 Biosun	Philips TL 20W/03 RS 1SL
**Light**	$T^{a}$ 4.000 °K	$T^{a}$ 6.500 °K	TL 20W/03 RS 1SL
**Manufacturer**	Philips, Consumer Lifestyle, Spain	Radium Lampenwerk GmbH, Germany	Philips, Consumer Lifestyle, Spain
**Emission**	34.8% of 400–500 nm	54.6% of 400–500 nm	84.3% of 400–500 nm
**Irradiation**	28.57 W/m^2^	93.46 W/m^2^	27.85 W/m^2^

## Data Availability

All data presented this study are available from the corresponding author, upon responsible request.
